# Prospects of Photonic Crystal Fiber as Physical Sensor: An Overview

**DOI:** 10.3390/s19030464

**Published:** 2019-01-23

**Authors:** Moutusi De, Tarun Kumar Gangopadhyay, Vinod Kumar Singh

**Affiliations:** 1 Indian Institute of Technology (Indian School of Mines), Department of Applied Physics, Fiber Optics Lab, Dhanbad 826004, India; vksingh@iitism.ac.in; 2 Fiber Optics and Photonics Division, CSIR-Central Glass and Ceramic Research Institute, CSIR, Kolkata 700032, India

**Keywords:** photonic crystal fiber, temperature sensors, pressure sensors, strain sensor, twist or torsion sensor, curvature or bend sensors, electromagnetic sensors, refractive index sensors

## Abstract

Photonic crystal fiber sensors have potential application in environmental monitoring, industry, biomedicine, food preservation, and many more. These sensors work based on advanced and flexible phototonic crystal fiber (PCF) structures, controlled light propagation for the measurement of amplitude, phase, polarization and wavelength of spectrum, and PCF-incorporated interferometry techniques. In this article various PCF-based physical sensors are summarized with the advancement of time based on reported works. Some physical PCF sensors are discussed based on solid core as well as hollow core structures, dual core fibers, liquid infiltrated structures, metal coated fibers, grating incorporated fibers. With the advancement of sensing technology the possibilities of temperature, pressure, strain, twist, curvature, electromagnetic field, and refractive index sensing are discussed. Also, limitations as well as possible solutions and future hopes are outlined.

## 1. Introduction

PCF was invented by invented by Russell and his colleagues at the end of 20th century [1]. From its invention PCF is showing its potential not only in low loss communication but also in many versatile and improved applications-sensing is one of them. Investigation of different physical parameters using photonic crystal fiber (PCF) is an integrative branch of optics as well as engineering. It successfully integrates fiber optics, structural engineering, electromagnetism, laser optics, the infiltration technique, optoelectronics, microelectronics, and material science. Photonics crystal fiber sensors are advantageous over other electrical and optical fiber sensing system in many aspects.

PCF has an advantageous geometry over standard optical fiber. Generally, PCF has either a hollow core or a solid core around which air holes are distributed in different patterns. Light is guided by the distribution of these air holes. Also, propagation of light can be manipulated by changing the distribution of air holes as well as with the environmental change [2,3,4]. This unique nature of PCF is drawing a lot of attention for its sensing applications from last two decades. PCF-based sensors became the focus of many research groups due to their high sensitivity, flexibility, small size, robustness, and that they can be used in many unfavorable situations. The small physical dimensions of PCF-based sensing probes make them suitable for attaching or inserting in a system. These sensing probes can be connected with the control system without the use of any wire. They can be used in a hazardous and noisy environment or high temperature, high voltage, high electromagnetic field, and explosive environments even for the purpose of remote sensing.

**Figure 1:**
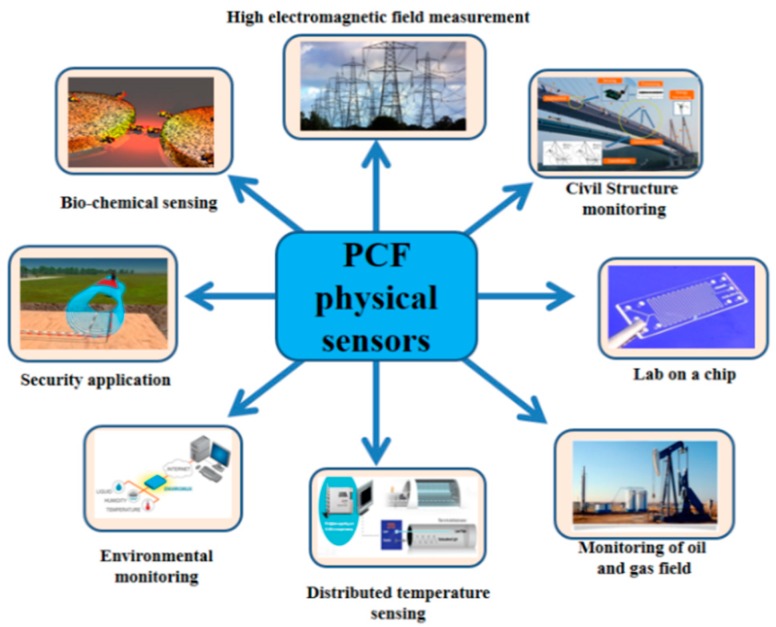
Applications of photonic crystal fiber (PCF) physical sensors.

Measurement of different physical parameters like, temperature, pressure, strain, twist or torsion, curvature or bend, and electromagnetic field is necessary for regular application, shown in Figure 1. In this article we tried to present an updated and compact description of PCF sensors applied or proposed for physical parameter sensing. It consists theoretical background of PCF, its wide range of applications for physical parameter measurement, current technologies as well as short comes and finally ended with concluding discussion.

## 2. Theoretical Framework of PCF

**Figure 2:**
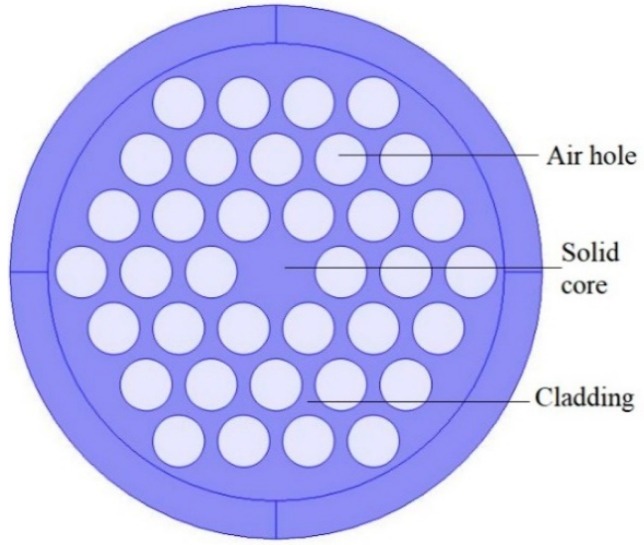
Diagram of solid core PCF.

**Figure 3:**
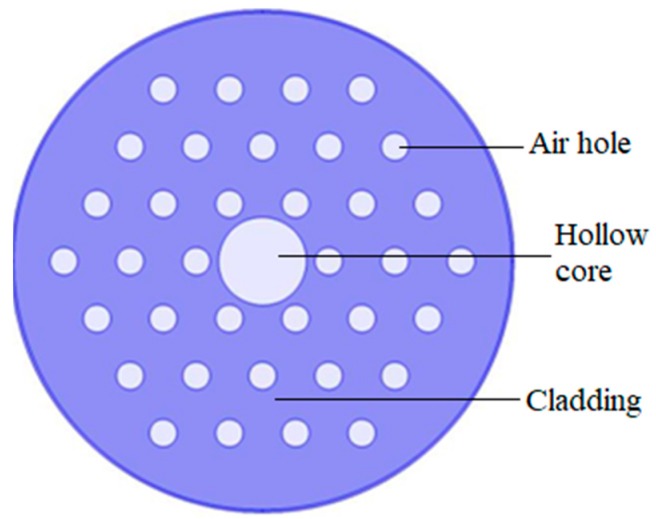
Diagram of hollow core PCF.

For a conventional optical fiber both the core and cladding both are solid. Generally cladding is pure silica and the core is doped glass-having a relatively high refractive index in comparison to cladding [5]. So, there is a positive refractive index difference between core and cladding. But in the case of PCF this refractive index difference is imposed by placing air holes in cladding. These air holes run throughout the length of the fiber and took an important role in guiding light through the core. Depending on the core nature PCF can be divided in two broad categories: solid core PCF and hollow core PCF. If the core is solid then it is solid core PCF (Figure 2). It has a positive refractive index difference between the core and cladding and it works based on the total internal reflection (TIR) phenomenon [2,6]. For hollow core PCF (Figure 3), the core is made of air and it has a negative refractive index difference between the core and cladding. It works based on photonic bad gap guiding mechanism [2,7].

PCF has many attractive properties compared to standard optical fibers like, highly birefringent [3,8], very low loss [9,10], endless single-mode propagation through a long wavelength range [1,11,12], highly nonlinear [13,14], dispersion tailoring [15,16], and large mode area [17,18]. These unique optical properties encourage researchers to use PCF not only in the field of communication [14,19,20,21] but also in spectroscopy [22], supercontinuum generation [23], nonlinear applications [24], Raman fiber laser [25,26], sensing [27], etc. Using the holey nature of PCF many sensors are proposed as well as fabricated with very high sensitivity [28,29]. PCF-based advanced physical sensors will be discussed in the following sections.

For an optical fiber, V-number and Numerical aperture (NA) are two important optical parameters. Endlessly single-mode (ESM) propagation of a PCF is decided by its V-number. Through a long wavelength range, the single-mode propagation capability of a PCF due to its exceptional cladding microstructure is defined as endlessly single-mode propagation. The V-number of a PCF can be calculated according to Mortensen et al. [11] using the following formula,

(1) $$V_{PCF}\left(\lambda \right)=\frac{2\pi \Delta }{\lambda }\sqrt{n_{core}^{2}\left(\lambda \right)-n_{eff}^{2}\left(\lambda \right)}$$

where Λ is pitch of PCF. From Equation (1) it can be observed that endlessly single-mode operation of a PCF depends on both its parameter and propagating wavelength. For PCF, the single-mode cut-off criteria is *V_PCF_* < π. Here, *n_eff_* is the effective refractive index of guided-mode of PCF and *n_core_* is refractive index of the core. *n_eff_* can be expressed as *n_eff_* = *β*/*k*_0_—where *β* is the propagation constant and *k*_0_ is free space propagation constant. Also *k*_0_ can be expressed as *k*_0_ = 2π/*λ*, where *λ* is the propagating wavelength [30].

Light gathering potential of an optical fiber is represent by numerical aperture. It is a dimensionless quantity. Large NA indicates more light gathering capability of a fiber. For PCF, NA can be calculated using the following formula [30],

(2) $$NA=\sqrt{n_{core}^{2}-n_{eff}^{2}}$$

From the very start, different kinds of PCFs are designed, analyzed by using commercial software as well as fabricated. Mostly, these analyzed works are based on either the finite element method or the finite difference time domain method. In both methods the whole structure is meshed in triangular pieces then the electromagnetic equations are applied in each section and the light guiding nature is studied. These techniques are very popular in computational electromagnetism [31,32,33]. They reduce losses during fabrication and enhance accuracy.

Many sensors are reported based on dual core PCF. Dual core PCF works based on mode coupling theory due to the coupling that four supermodes (*x* even, *y* even, *x* odd, and *y* odd modes) generate. For one coupling length power transfer completely from one core to another. At output optical power intensity can be calculated as

(3) $$I\left(\lambda \right)=1-\cos\left\{\frac{\pi }{\lambda }\left(\Delta n_{x}+\Delta n_{y}\right)\right\}\cdot \cos\left\{\frac{\pi }{\lambda }\left(\Delta n_{x}-\Delta n_{y}\right)\right\}$$

Here, Δ*n_i_* with *i* = *x*,*y* is effective refractive index difference of *x* polarized even-odd mode and *y* polarized even-odd mode. Output intensity curve is sinusoidal in nature. Sensitivity of a dual core PCF consisting probe can be calculated from the output transmission peak shift with changing environment [34,35].

## 3. Overview of PCF Physical Sensors

PCF-based sensors are advantageous over standard optical fiber sensors in many aspects. They not only have great design flexibility but also their holey internal structure can be filled with analyte so that a controlled interaction can take place between propagating light and the analyte sample [36]. This greatly enhances the sensitivity of fiber optic sensors as well as opens up a new direction for making advanced portable sensors. PCF sensors have a wide range of applications. Measurement of different physical parameters like temperature, pressure, strain, twist, torsion, curvature, bend, and electromagnetic field are a few of them. Observation as well as control of these parameters are really important in many daily life applications including civil structural health monitoring [37]. PCF-based physical sensors are gaining a lot of attention due to their in situ and remote sensing capabilities; immunity from the hazardous environments of high electromagnetic field and high voltage; and biomedical sensing capability [37,38]. At the very beginning main focus was on fabricating PCF-based sensors using different interferometry techniques. However, with time the focus has shifted toward the design and fabrication of new PCF structures with advanced optical properties and their application for making sensors. Evolution of different physical sensors are discussed in the following sections.

### 3.1. Temperature Sensors

**Figure 4:**
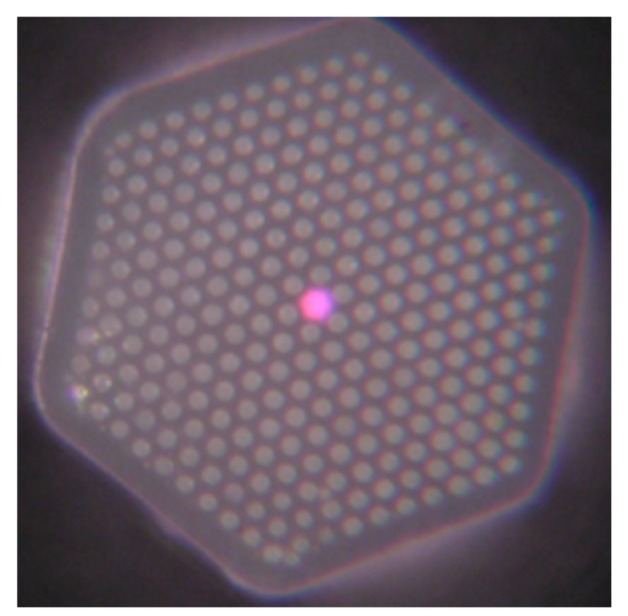
End-face of the liquid crystal infiltrated PCF for the measurement of temperature and electric fields (Reproduced from [40], with the permission of IOP science publishing).

**Figure 5:**
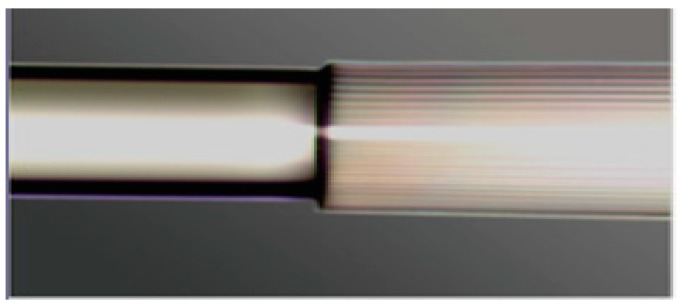
Microscope image of PCF-MMF spliced probe based on Quantum dot nanocoatings used for temperature sensing (Reproduced from [41], with the permission of Hindwai publishing).

**Figure 6:**
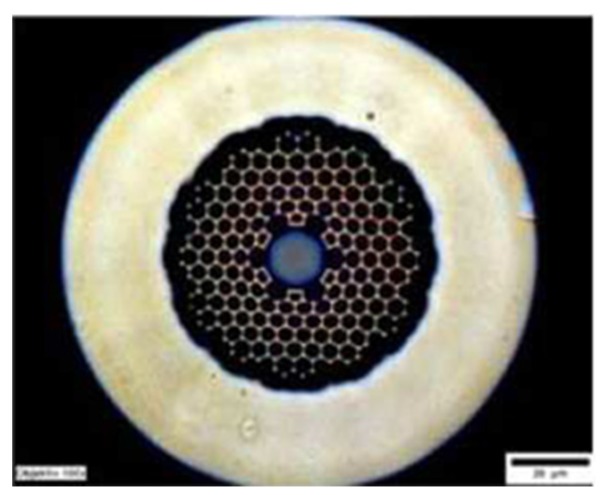
Cross-section image of a 7-cell hollow core PCF used for temperature measurement (Reproduced from [28], with the permission of OSA publishing).

**Figure 7:**
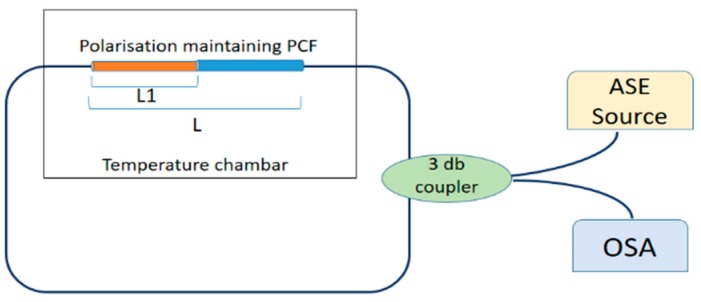
Schematic diagram of the optical fiber Sagnac interferometer-based temperature sensor; here L is the full length and L_1_ is the infiltration length of PM-PCF in this fiber loop (Figure courtesy of reference [42]).

Temperature measurement is an important physical parameter for all fields of technical activities, industrial stages of production and maintenance and also in medical treatments. The invention of fiber optic sensors for temperature measurement was a great breakthrough representing a viable alternative to the use of electronic sensors. These sensors can be made with multimode fiber (MMF), single-mode fiber (SMF), and enhances the sensitivity of measurement using a laser source with optoelectronics. The most practical advantage is to use fiber-based temperature sensors in the field of applications where electromagnetic interference (EMI) and RF are vital obstructions to the use of electronic sensors. PCF-based sensors are also useful in aerospace, defense, the chemical industry, semiconductor industry, civil engineering applications, turbine areas, and many more. In the field of these sensors, Zhu et al. fabricated, as well as demonstrated [39], strain-insensitive and high-temperature long-period gratings sensors inscribed in solid core PCF having sensitivity of 10.9 pm/°C from 24 °C to 992 °C illuminated by CO_2_ laser at 1299.59 nm wavelength. A hexagonal solid core PCF sensor was reported [40] with sensitivity 7 nm/°C when air holes were filled with 1550 nm liquid crystal (Figure 4) for the measurement of temperature and electric fields. A layer-by-layer quantum dot nanocoatings on the inner holes of PCF (LMA-20) spliced with MMF is experimentally demonstrated [41] in the temperature range of 40 °C to 70 °C with wavelength sensitivity 0.1451 nm/°C (Figure 5). A 7-cell HCPCF sensor spliced with a SMF was reported previously [28] with sensitivity −7.1 pm/°C (Figure 6). Another selectively filled polarization-maintaining PCF (PMPCF) temperature sensor based on the Sagnac interferometer was reported [42], in which L_1_ is the infiltration length and L is the total length of PM-PCF inside the fiber loop as shown in Figure 7. It has sensitivity 2.58 nm/°C for the 11.7 cm-long fiber as measured from transmission wavelength shift.

**Figure 8:**
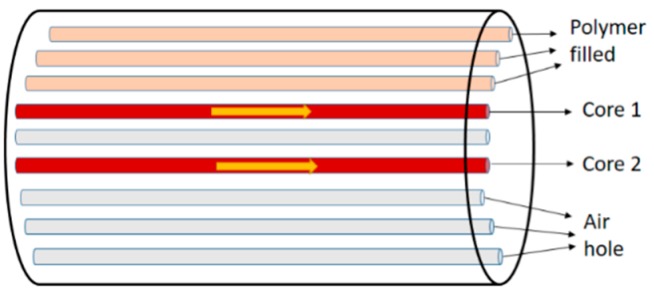
Schematic diagram of the half-filled twin core PCF after the selective polymer filling. It was used for temperature sensing (Figure curtsy from [44] article).

**Figure 9:**
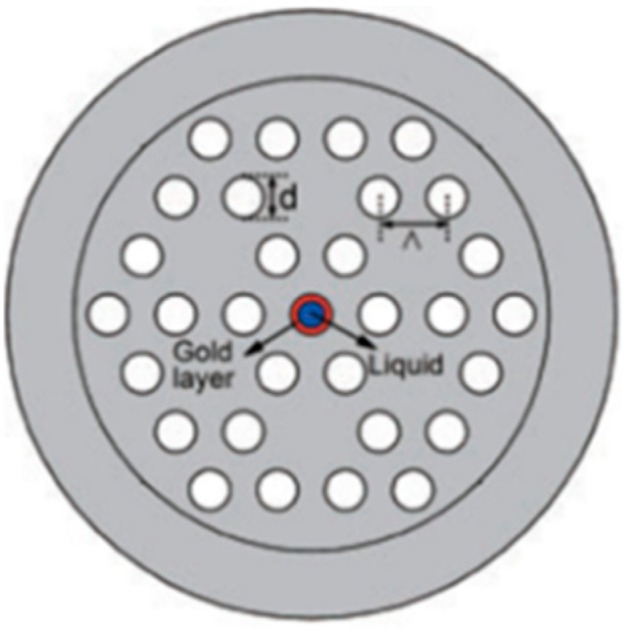
Cross-section of the proposed nanoscale gold layer incorporated PCF for temperature sensing (Reproduced from [31], with the permission of IOP Science publishing).

A relatively new structured PCF temperature sensor [43] was demonstrated when a standard Si wafers was attached to the facet of a standard single-mode optical fiber. It acts as a tip sensor with a sensitivity of 0.11 nm/°C in the 100 °C to 700 °C temperature range. Another temperature sensor was fabricated [44] based on Mach–Zehnder interference and dual core PCF with selectively polymer-filled air holes with high sensitivity 1.595 nm/°C (Figure 8). A surface plasmon resonance based PCF temperature sensor with nanoscale gold coating of the central air hole was reported [31] with sensitivity −2.15 nm/°C (Figure 9). Fully and partially ethanol-filled photonic bandgap fibers spliced between standard SMF was reported [45] having temperature sensitivity −292 pm/°C and −120 pm/°C for fully and partially filled ethanol, respectively. Few more temperature sensors are presented in Table 1.

### 3.2. Pressure Sensors

**Figure 10:**
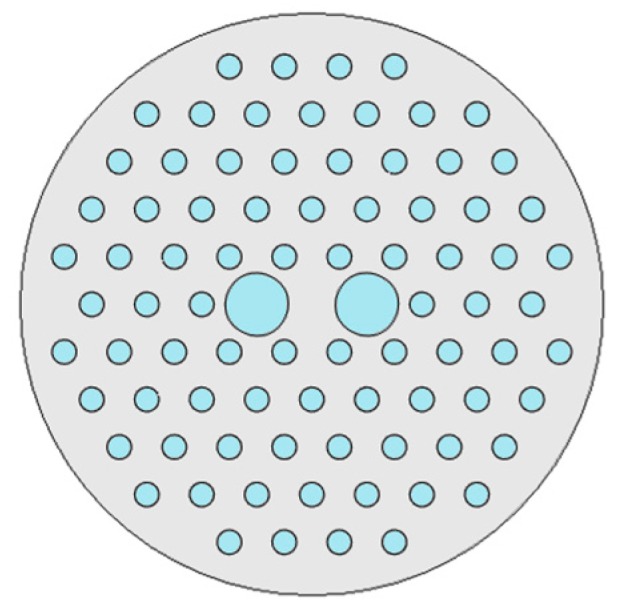
Cross-section of polarization-maintaining PCF 1550-01 used for pressure sensing (Figure curtsy from [56] article).

**Figure 11:**
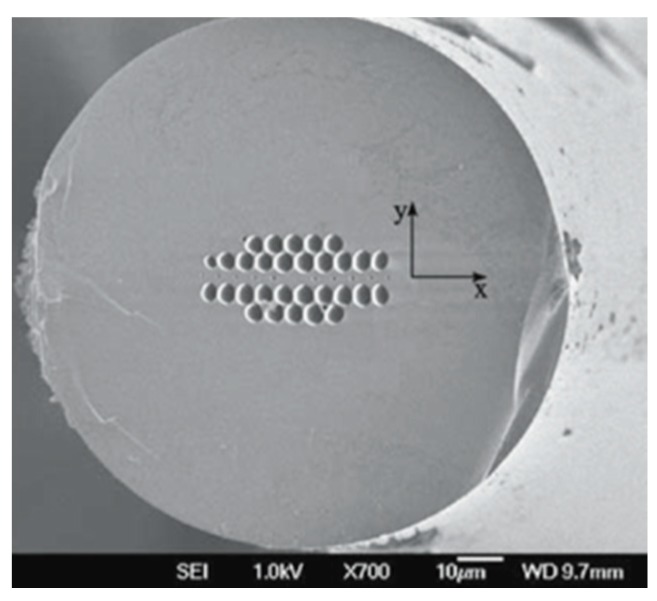
SEM image of the high birefringent index-guided PCF used for the measurement of polarimetric sensitivity to hydrostatic pressure (Reproduced from [57], with the permission of Springer publishing).

**Figure 12:**
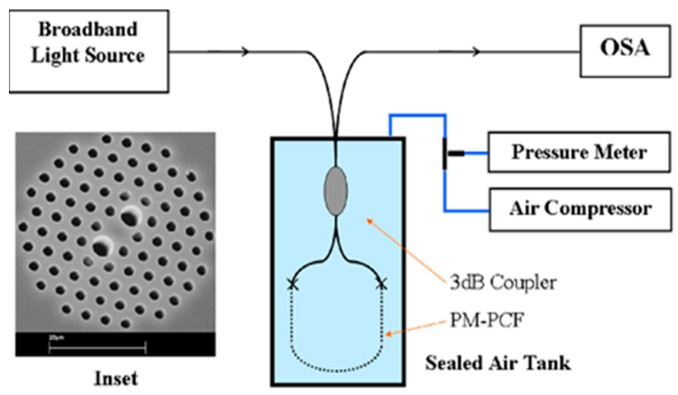
Schematic diagram of the proposed pressure sensor constructed with a polarization-maintaining PCF-based Sagnac interferometer. Inset consist used a polarization-maintaining PCF (Reproduced from [58], with the permission of OSA publishing).

**Figure 13:**
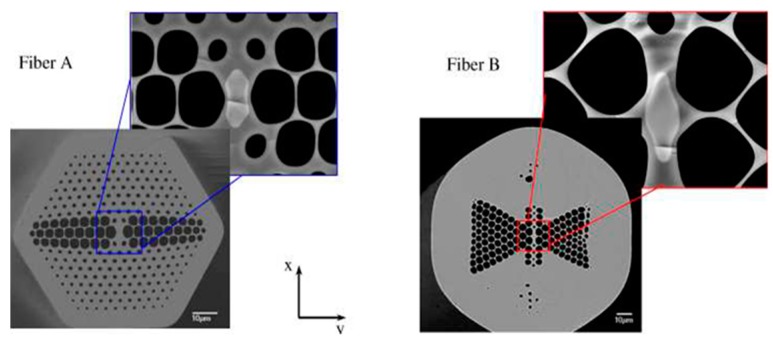
SEM images of two birefringent microstructured fabricated fibers with enhanced sensitivity for the measurement of hydrostatic pressure (Reproduced from [59], with the permission of OSA publishing).

**Figure 14:**
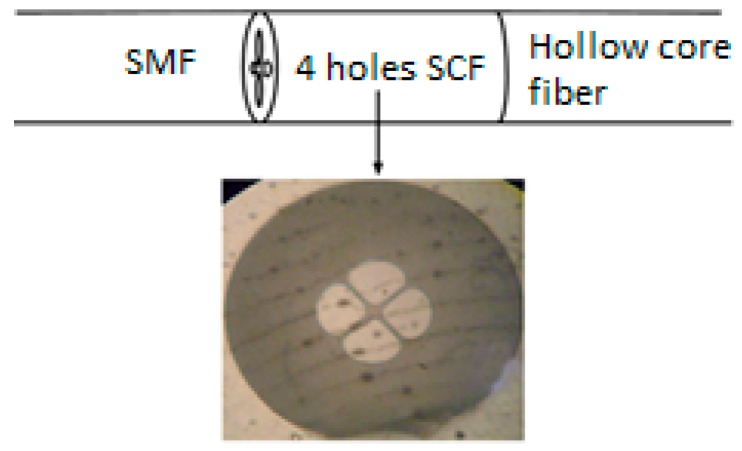
Schematic of the Fabry-Perot cavity as a sensing head with cross-sectional microscopic image; the four holes suspended core fiber section forms the Fabry-Perot cavity (Reproduced from [60], with the permission of Elsevier publishing).

**Figure 15:**
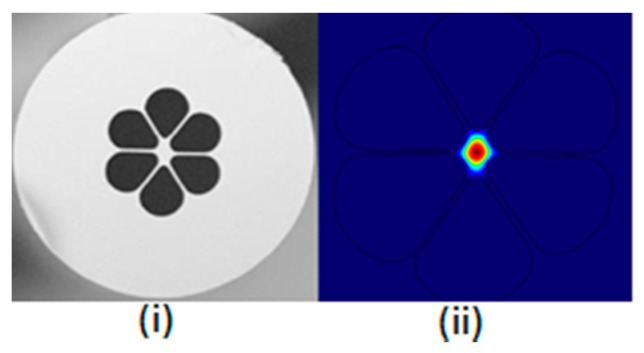
SEM micrograph of the cross-section of solid core polarization-maintaining PCF and its optical mode profile (Reproduced from [32], with the permission of Elsevier publishing).

Pressure is an important physical quantity when observing many environmental phenomena in precision application areas as well as monitoring many industrial processes in spite of the harsh environment. Due to the good compatibility of fiber pressure sensors with human and other animal bodies they can be used in medical diagnosis purpose also. PCF-based pressure sensors can be used in measuring human body fluid pressure. These sensors are also suitable in measuring temperature and pressure under water. In 2005, a polarization-maintaining PCF PM-1550-01-based pressure sensor was developed [56] by Blaze photonics (Figure 10). A hydrostatic pressure sensor was reported [57] with highly birefringent PCF and sensitivity −10 rad/MPa.m at 1.44 μm wavelength (Figure 11). Then a compact pressure sensor was developed [58] using Sagnac interference with a polarization-maintaining PCF with sensitivity 3.42 nm/MPa for a 58.4 cm long fiber (Figure 12). Another highly birefringent hydrostatic pressure sensor was demonstrated [59] theoretically as well as experimentally having sensitivity more than −43 rad/MPa.m at 1.55 μm wavelength (Figure 13). A sensor was reported [60] using a Fabry–Pérot cavity in which a microstructure fiber is spliced between SMF and hollow-core fiber with pressure sensitivity −4.68 × 10^−5^ nm/psi (Figure 14). A pore water pressure sensor was reported [32] with a six-hole suspended-core PM-PCF-based Sagnac interferometer having sensitivity 254.75 kPa/nm for a 100 cm long fiber (Figure 15). Excluding the sensors listed above a few more pressure sensors are presented in Table 2.

### 3.3. Strain Sensor

Strain measurement is a necessary requirement in industrial application and precision control system. Fiber optics-based strain sensors can be used in earthquake damage detection, in defense applications, monitoring telecommunication cables during temperature variation, process control, load control on important bridges & structures, fire detection, etc. These sensors have important applications in civil engineering: in bridge monitoring, welding residual stresses monitoring, observation of old heritage buildings, pipeline monitoring, and other structural health monitoring. An endlessly single-mode PCF-based cable consisting a long period grating has been fabricated [66] using a spatially periodic electric arc discharge technique. It has strain sensitivity −2.0 pm/µε. Using a highly birefringent PCF loop mirror coated with acrylate material a strain sensor was reported [67] with enhanced sensitivity of 1.21 pm/µε. The length of the sensing head for this sensor was 380 mm. A hollow-core photonic band gap fiber based Fabry–Pérot (FP) interferometric strain sensor was reported [68] having FP cavity in the order of millimeters and fabricated by the simple techniques of cleaving and fusion splicing. The sensitivity of the sensor is 1.55 pm/µε at the wavelength of 1550 nm and suitable for a wide range of applications.

**Figure 16:**
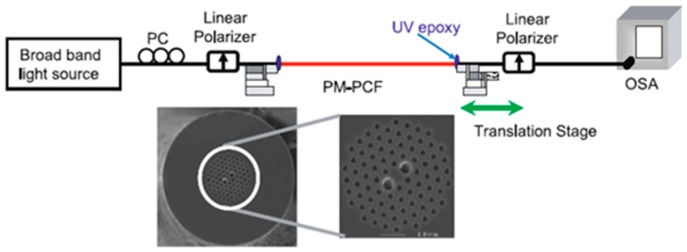
Experimental setup of the proposed temperature-insensitive polarization-maintaining PCF strain sensor and SEM image of the fiber (Reproduced from [69], with the permission of Springer publishing).

**Figure 17:**
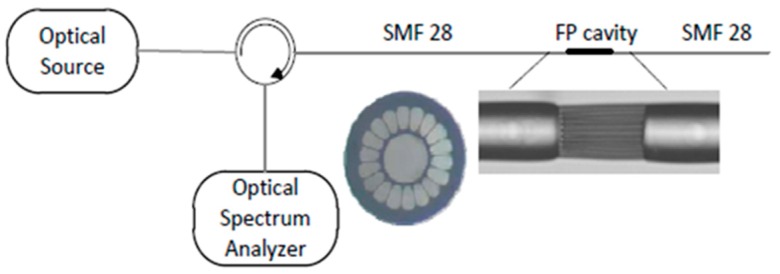
Schematic diagram of the experimental setup and SEM image of the hollow core PCF and sensing probe (Reproduced from [70], with the permission of OSA publishing).

**Figure 18:**
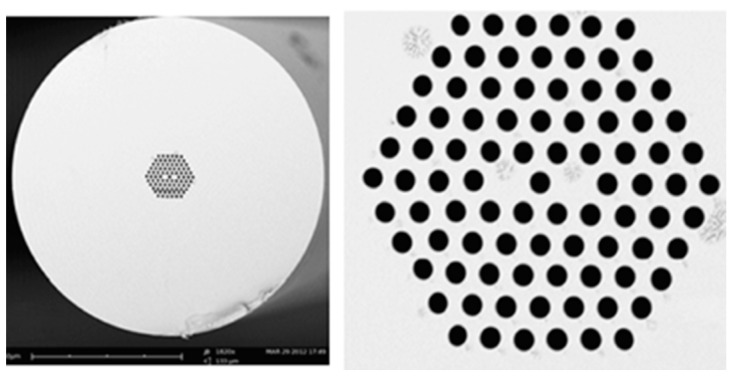
SEM image of twine core PCF. Ten centimeters of this fiber were used to form an in-fiber Mach–Zehnder interferometer for strain measurement (Reproduced from [71], with the permission of Elsevier publishing).

A low loss PM-PCF-based birefringent interferometer strain sensor was reported [69] with strain sensitivity 1.3 pm/με in a strain range from 0 με to 1600 με (Figure 16). A F-P cavity having length 207 μm was fabricated by splicing a hollow-core ring PCF between two standard SMF and using this a strain sensor was fabricated [70] with sensitivity 15.4 pm/µε for a FP cavity of 13 µm length (Figure 17). A strain sensor was reported [71] using a dual-core PCF-based Mach–Zehnder interferometer having sensitivity −0.31 pm/µε within a range 0 με to 4000 µε (Figure 18). A multi components interferometer based on partially filled dual-core PCF was demonstrated [72] in which cladding air holes surrounding of core *a* were blocked by glue and air holes surrounding of core *b* were kept open. It has sensitivity −2.08 pm/µε. A few more strain sensors are presented in Table 3.

### 3.4. Twist or Torsion Sensor

Torsion is an important parameter that has to measure for different civil structure for safety purpose. For immunity against harsh environment, light weight, small size, and high shock survivability are required and these types of sensors are attracting attention due to their suitability for industrial use. A single-mode PCF was demonstrated [79] as a torsion sensor by including stress induced mechanical long-period grating. The sensitivity of this sensor is 0.73 nm/2π. A two-linearly polarized mode operation in a ultrahigh birefringent photonic crystal fiber-based twist sensor was reported [80] having sensitivity of 8.25/°C with resolution ~2.7° for the range 90–270°.

**Figure 19:**
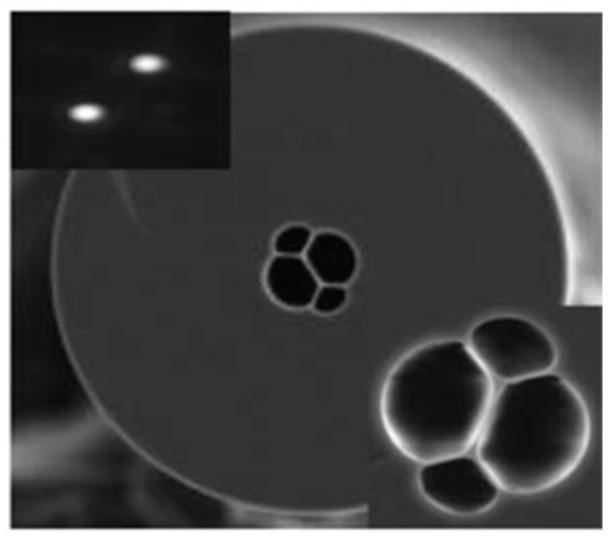
A fiber loop mirror configuration is formed using this suspended twin-core fiber for torsion measurement (Reproduced from [81], with the permission of OSA publishing).

**Figure 20:**
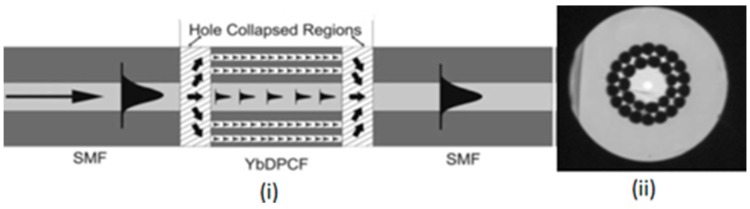
(**i**) Schematic diagram of the three beam path MZI used for torsion sensing and (**ii**) image of the Yb-d-DPCF cross-section (Reproduced from [85], with the permission of Wiley publishing).

A suspended twin-core fiber based loop mirror configuration was demonstrated [81] as a torsion sensor having sensitivity 5.1 × 10^−4^/°C (Figure 19). Another sensor was reported [82] using Hi-Bi PCF-based Sagnac interferometer having sensitivity ~0.06 nm/°C. A torsion sensor was reported [83] using side leakage PCF with sensitivity 0.9354 nm/°C over a range of 0 to 90 in both clock wise and anticlockwise direction. Thereafter, a solid core low birefringence PCF (LMA-10)-based Sagnac interferometer using torsion sensor was proposed [84] with sensitivity 1.00 nm/°C and resolution 0.01°. A three-beam path Mach–Zehnder interferometer was formed [85] by fusion splicing a piece of double ytterbium-doped double-cladding PCF between two segments of SMF to fabricate torsional sensor with sensitivity 0.001 nm/°C (Figure 20).

### 3.5. Curvature or Bend Sensors

**Figure 21:**
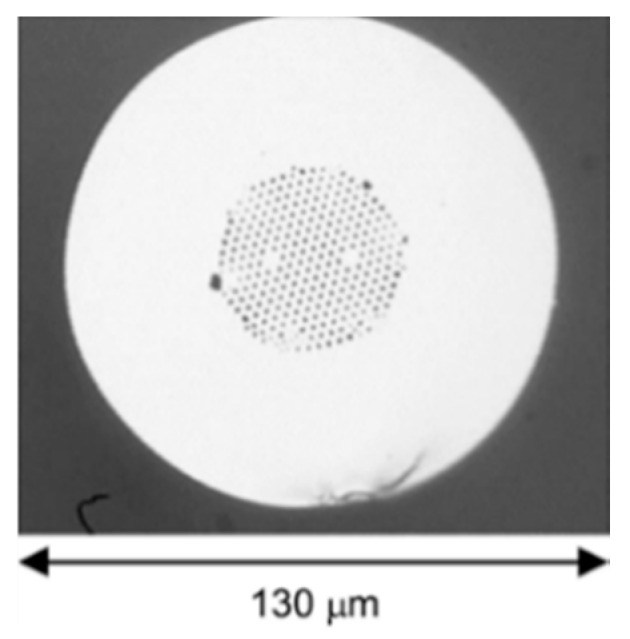
Microscopic image of the curved end face of a two core PCF. These two cores help in forming a two-beam interferometer and curvature is measured using the phase difference between two beams (Reproduced from [86], with the permission of Elsevier publishing).

**Figure 22:**
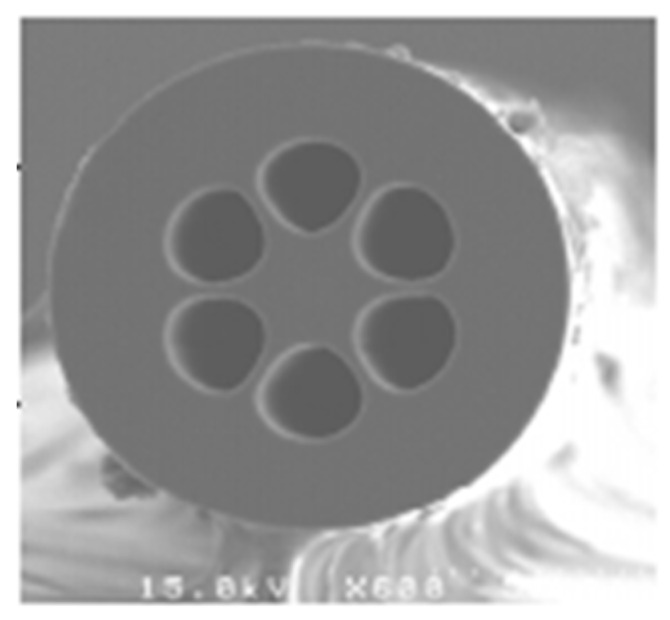
In this hollow fiber long-period fiber grating is inscribed and used for bending properties measurement (Reproduced from [87], with the permission of OSA publishing).

**Figure 23:**
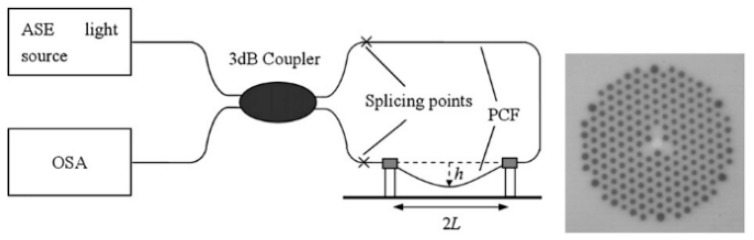
Demonstrated Sagnac loop using a low birefringence PCF for curvature measurement and the micrograph of that PCF (Reproduced from [84], with the permission of Elsevier publishing).

**Figure 24:**
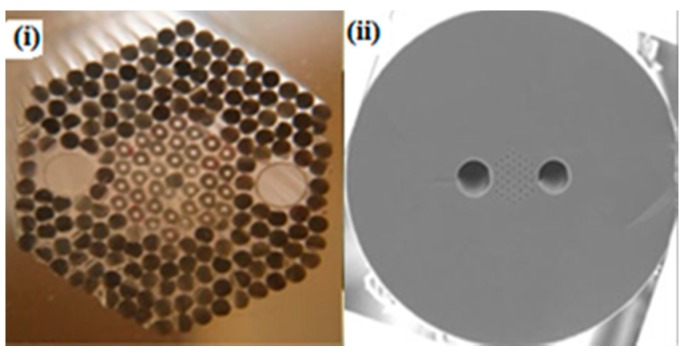
Image of two large air holes consisting PCF; cross-section of (**i**) the preform and (**ii**) fabricated high-birefringent fiber. It is used for the demonstration of a curvature sensor (Reproduced from [85], with the permission of IOP Science publishing).

Curvature is also an important parameter in structural health monitoring. Curvature sensors are useful in robot making, in medical tooth root canaling treatment, in artificial organs, etc. In 2001 a two core PCF was used [86] to make a two-beam interferometer to measure its phase change curvature variation and sensitivity 127 rad/rad (Figure 21). A long-period fiber grating incorporated into a holey fiber was reported as a bending sensor considering its axial rotation angle [87]. This sensor shows a shift of the central wavelength into the shorter wavelength for bending curvature higher than 4 m^−1^ also the bending sensitivity change by rotational orientation (Figure 22). A two-asymmetric hole region consisting of a highly birefringent PCF inserted into a Sagnac interferometer was demonstrated as a curvature sensor [88]. It is able to work in a curvature range of 0.6 to 5 m^−1^. A curvature sensor was demonstrated [89] with a low-birefringence PCF-based Sagnac loop, consisting of a 40 cm-long PCF having curvature measurement sensitivity of −0.337 nm in the range of 0–9.92 m^−1^ (Figure 23). A curvature sensor was reported by Hwang et al. [90] using novel PCF of high birefringence based Sagnac interferometer. Its sensitivity depends on the bending direction. Obtained sensitivity is −1.87 nm/m^−1^ and 1.24 nm/m^−1^ for parallel and perpendicular bending, respectively, to the large air hole axis near 1480 nm wavelength (Figure 24).

**Figure 25:**
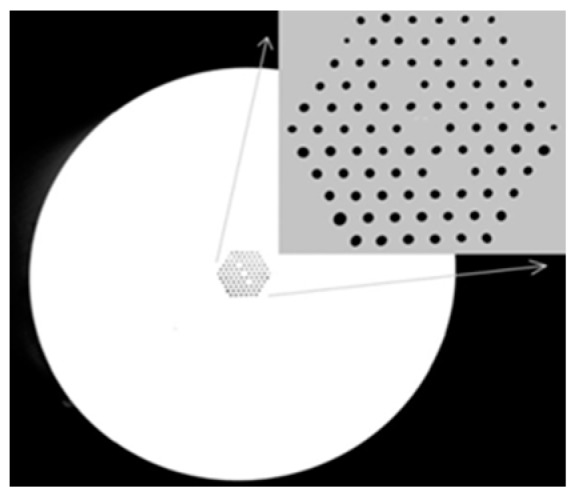
Cross-section of the PCF with three cores which is used for making intensity curvature sensor (Reproduced from [91], with the permission of IOP Elsevier publishing).

**Figure 26:**
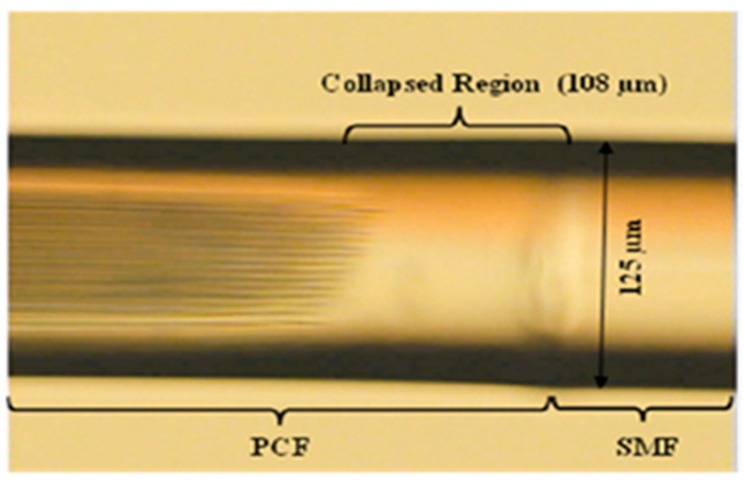
Micrograph of splicing region between PCF and SMF. Using it Mach–Zehnder interferometer is formed for curvature measurement (Reproduced from [92], with the permission of IOP Elsevier publishing).

**Figure 27:**
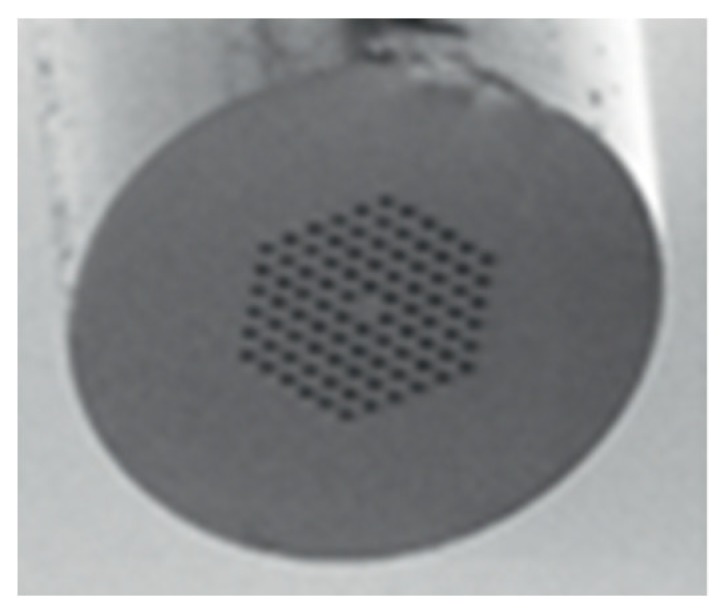
SEM image of PCF used for long-period gratings inscription and structural health monitoring (Reproduced from [89], with the permission of Elsevier publishing).

**Figure 28:**
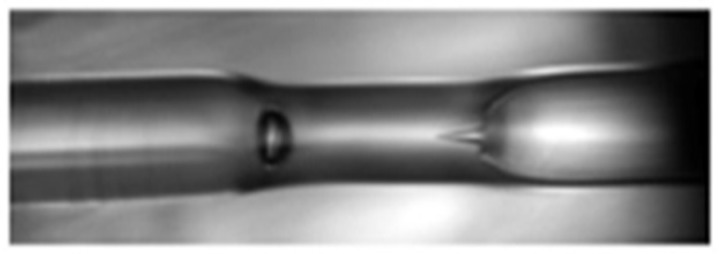
A micro-air cavity is formed by splicing hollow core PCF and SMF for curvature measurement (Reproduced from [90] with the permission of IOP science publishing).

Then a three-coupled core consisting of a PCF-based curvature sensor was reported [91] with a maximum curvature sensitivity of 2.0 dB/m^−1^ for the curvature range 0 to 2.8 m^−1^ (Figure 25). A curvature sensor was demonstrated [92] using a tapered PCF collapsed with SMF-based Mach-Zehnder interferometer with sensitivity 8.35 dB/m^−1^ in between curvature 0.87 and 1.34 m^−1^ with resolution 0.0012 m^−1^ (Figure 26). Another curvature sensor was reported [93] with a hollow core PCF-based Sagnac interferometer having sensitivity 0.232 nm/m^−1^ in the curvature range of 0 to 9.9 m^−1^. A cladding modes analyzation-based long-period gratings PCF curvature sensor was proposed [94] with sensitivity ~20 nm for 1550 nm wavelength for curvature range 0 to 2 m^−1^ (Figure 27). A microcavity curvature sensor was manufactured [95] by splicing a hollow core PCF at the end of a SMF. Its maximum sensitivity was found 10.4 dB/m^−1^ for the curvature range 0 to 1 m^−1^ with a second taper diameter of 18 μm (Figure 28).

### 3.6. Electromagnetic Sensors

**Figure 29:**
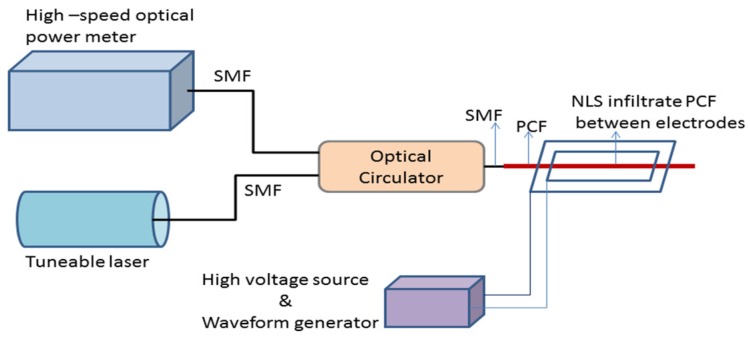
Schematic of the experimental setup to study reflected power response of the nematic liquid crystal (NLS) infiltrated PCF probe for the measurement of external electric field intensity (Reproduced from [96], with the permission of OSA publishing).

**Figure 30:**
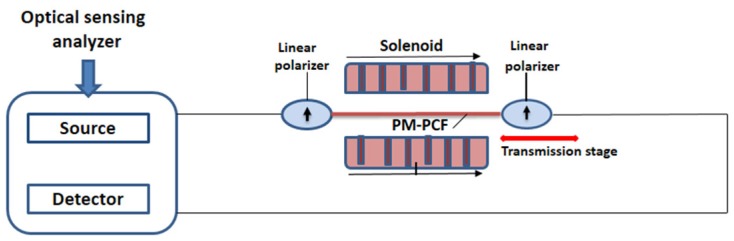
Experimental set up for magnetic field detection using polarization-maintaining PCF filled with Fe_3_O_4_ nanofluid (Reproduced from [97], with the permission of AIP publishing).

Electromagnetic field and associated force is one of the fundamental forces of nature. It creates strong and detectable for high electricity consuming objects which is harmful for leaving beings but this field is not detectable by the sense organs. So sensing of this field as well as its current in many cases is an important task. In electric power industry and other places presence of metal may influence the electromagnetic field measurement. So, fiber optics sensors are suitable for the same. Also, the properties of fiber for remote sensing are small size, nonconducting nature, and immunity to electromagnetic interference representing them as a suitable candidate in making electromagnetic sensors based on PCF. Among the various reported electromagnetic sensors some of them are discussed here. At the early stage a solid core PCF filled with liquid crystal (LC) was demonstrated [40] as an electric field sensor based on the orientation of the LC molecules with the changing of an applied electric field. Then moving a few steps forward based on this LC (MDA-05-2782) filling in a PCF (LMA-8) a sensor probe was reported [96] for measuring high electric field intensity with sensitivity ~10.1 dB/kV rms/mm for the electric field intensity range 2.35–4.95 kV rms/mm and resolution ~1 V rms/mm for in-line type transmitted mode (Figure 29). In the same year a magnetic field sensor was demonstrated [97] on a PM-PCF, by filling its cladding air holes with Fe_3_O_4_ nanofluid. This sensor has sensitivity 242 pm/mT for the concentration of the fluid 0.6 mg/mL (Figure 30).

**Figure 31:**
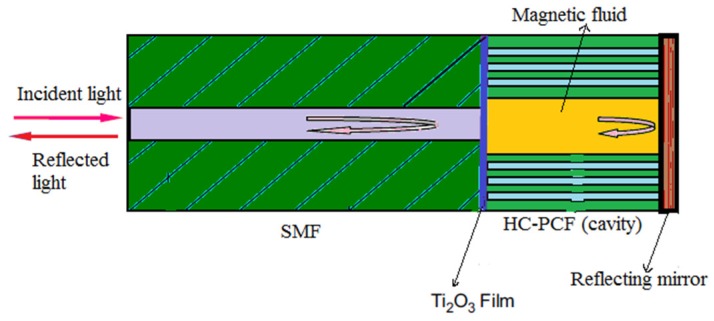
Schematic diagram of magnetic fluid filled hollow core PCF Fabry-Perot cavity sensor (Reproduced from [98], with the permission of Elsevier publishing).

**Figure 32:**
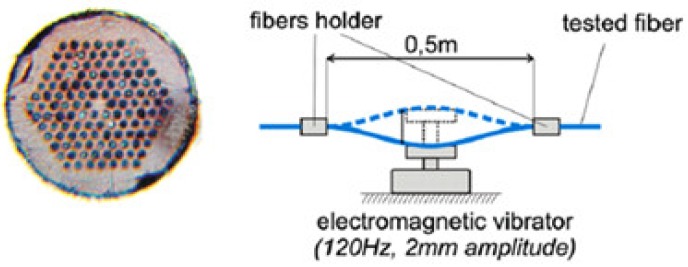
Cross-section of tested PCF and set up to measure current using electromagnetic vibration (Reproduced from [99], with the permission of author).

Then using hollow core PCF which forms a FP cavity, core of which is filled with water-based CdFe_2_O_4_ as the magnetic fluid, a magnetic field sensor was proposed [98] with sensitivity 33 pm/Oe for a very small sensor probe of 200 µm (Figure 31). A glass core PCF-based current sensor using electromagnetic vibration was reported [99] in the same period (Figure 32).

**Figure 33:**
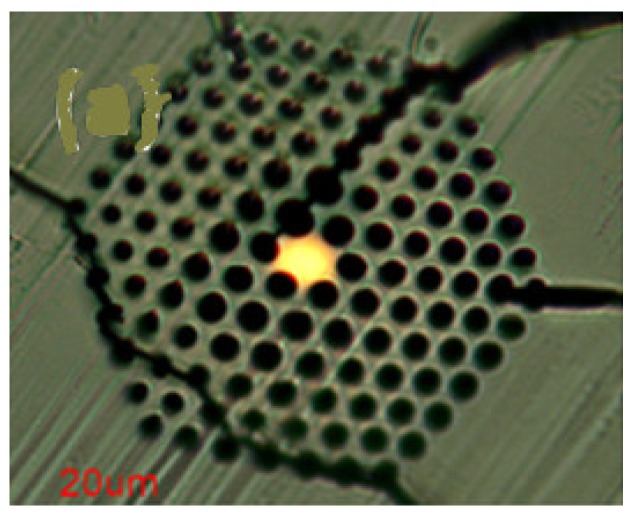
Microscopic image of the ferrofluid infiltrated microstructured polymer optical fiber. It is used for the measurement of magneto-driven optical loss effect of the sensor (Reproduced from [100], with the permission of AIP publishing).

**Figure 34:**
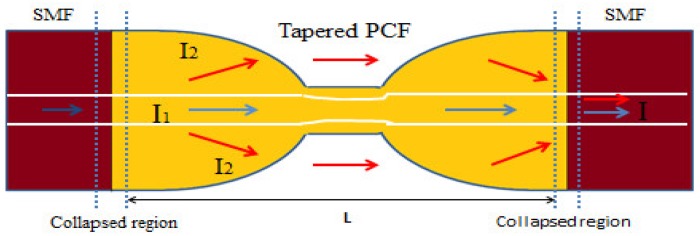
Schematic diagram of the Mach-Zehnder interferometer structure based on tapered PCF coated with ferrofluid for the magnetic field intensity measurement (Figure courtesy from the reference [101]).

**Figure 35:**
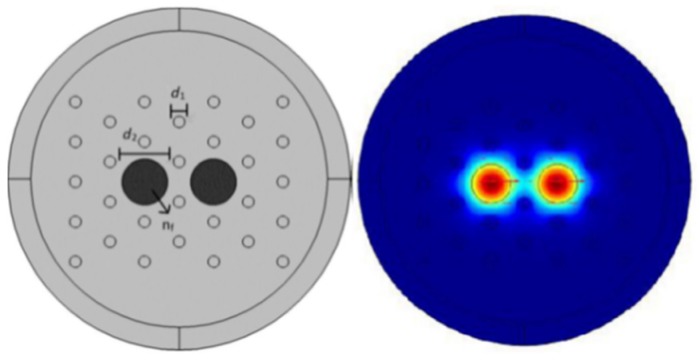
Cross-section of the designed magnetic fluids infiltrated dual core PCF magnetic field sensor and mode field pattern. This probe is useful for high magnetic field measurement (Reproduced from [34], with the permission of SPIE publishing).

**Figure 36:**
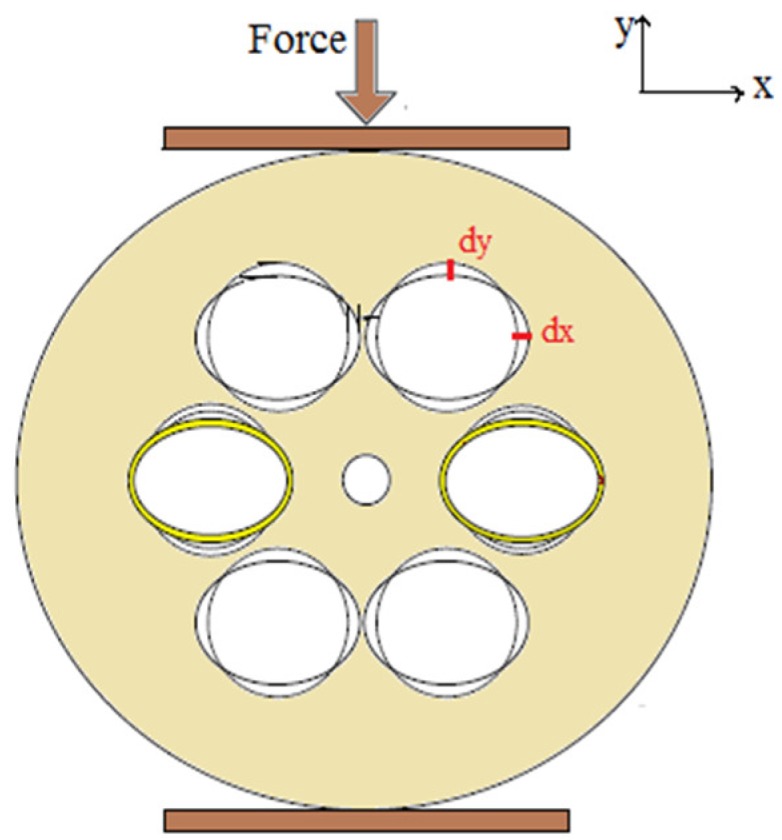
Spectral response of this selectively magnetic fluid filled and two cascaded gold layer incorporated PCF is studied in the presence of external applied force for the measurement of external magnetic field (Reproduced from [102], with the permission of Elsevier publishing).

A loss based ferrofluid (EMG905) infiltrated microstructured polymer optical fiber (MPOF) magnetic field sensor was demonstrated [100] which can measure the magnetic field change up to 2000 gauss for the magnetic field perpendicular to the fiber axis. The refractive index of the ferrofluid changes per magnetic field as ~1 × 10^−3^/100 G (Figure 33). A tapered PCF coated with ferrofluid (water-based ferrofluid EMG507, Ferrotec) was reported [101] as a magnetic field sensor. It was made by a tapered PCF spliced between two SMF. It has sensitivity 16.04 pm/G for the magnetic field range 100 to 600 G with resolution 0.62 G (Figure 34). Then a magnetic field sensing probe was proposed [34] which consists of a dual core PCF and both the cores are filled with Fe_3_O_4_ magnetic fluid. These two cores behave as two separate wave guides and mode coupling takes place between them. Based on mode coupling different high magnetic field can be identified from spectral shift. This probe has sensitivity 305.8 pm/Oe (Figure 35). Very recently a SPR technique was combined with PCF for the purpose of magnetic field detection in which two parts of gold-layer-filled PCF are joined together to achieve a localized SPR effect [102]. Cladding air holes of the two PCF segments were filled selectively with magnetic fluid and force was applied on one of it (Figure 36). It gives some new way to think about SPR based magnetic field sensors. Recently Yin et al. demonstrated [103] a nanomagnetic fluid filled double clad PCF-based magnetometer which is working on a modal interference mechanism and has a sensitivity 114.5 pm/mT. Also a polarization maintaining PCF incorporated Sagnac interferometer was proposed [104] for magnetic field detection. Water based nanoparticles Fe_3_O_4_ are infiltrate into the fiber. Refractive index changing property of magnetic fluid with changing magnetic field is used to study its two dip wavelengths shift with increasing magnetic field. It has sensitivity 384 pm/Oe in the detection range of 410 to 600 Oe. Recently, De et al. proposed a square lattice dual core photonic crystal fiber based magnetic field sensor with sensitivity 799.07 pm/Oe for magnetic field variation form 89.9 Oe to 271.0 Oe [105].

### 3.7. Refractive Index Sensors

**Figure 37:**
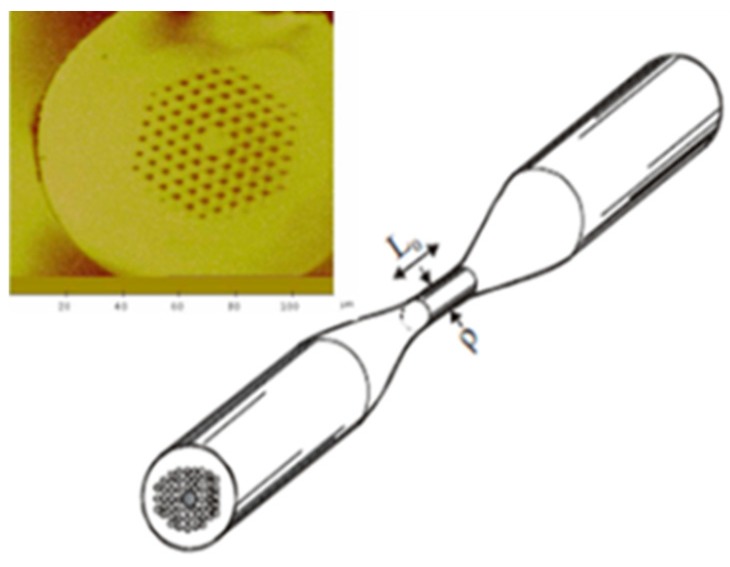
Cross-section of an untapered PCF and illustration of a uniform waist tapered PCF used for refractive index sensing. L_0_ is the tapper length and ρ tapper waist diameter (Reproduced from [106], with the permission of OSA publishing).

**Figure 38:**
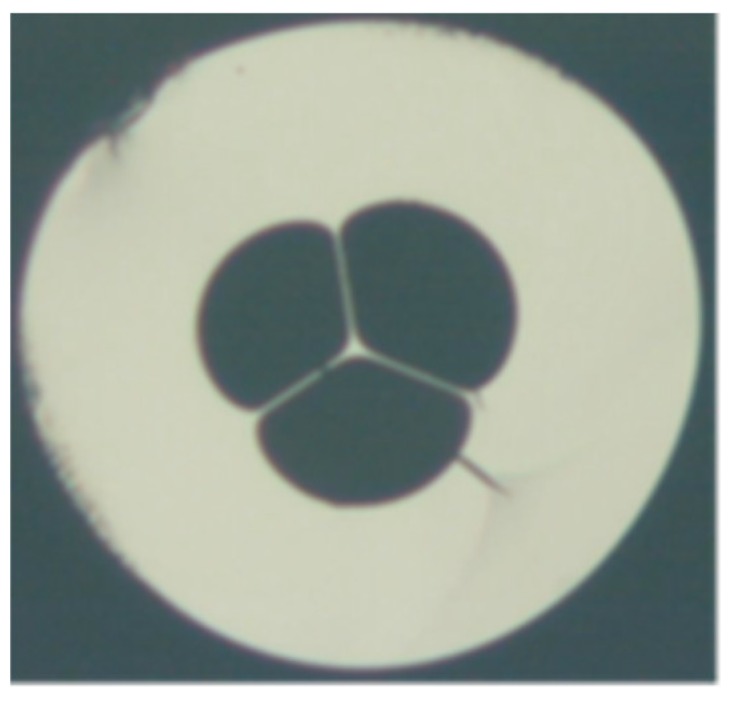
Microscope image of the three holes PCF with suspended Ge-doped silica core. It is combined with fiber Bragg grating and holes are filled with analyte then used as refractive index sensor (Reproduced from [107], with the permission of OSA publishing).

**Figure 39:**
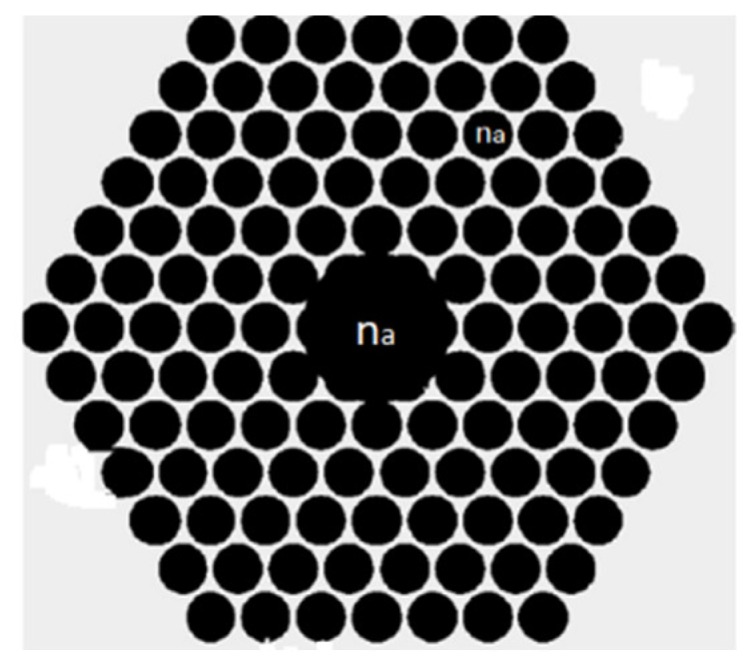
Schematic diagram of the photonic band gap fiber with hollow core. It is filled with tested analyte (n_a_) for refractive index measurement (Reproduced from [108], with the permission of Elsevier publishing).

Refractive index is an important basic physical parameter. In situ measurement of it helps to identify a material in many practical fields, like, chemical industry, gas and oil field industry, food processing and quality control industry, to check the adulteration level in liquid, for the identification of biomolecules, etc. At the beginning commercially available LMA PCFs were the main point of interest of many research groups for the development of PCF sensors. In 2005 a LMA-tapered holey fiber containing collapsed air hole refractive index sensor was experimentally demonstrated by Minkovich et al. [106] with a resolution around 1 × 10^−5^ for a refractive index higher than 1.44 (Figure 37). A combination of three-hole microstructured optical fiber and Fiber Bragg Grating (FBG) was reported [107] for refractive index sensing. Fiber Bragg grating (FBG) was written in the suspended Ge-doped silica core. It has a resolution of 3 × 10^−5^ and 6 × 10^−5^ for refractive index 1.33 and 1.40 (Figure 38). In 2007 Sun et al. reported a HC-PCF-based refractive index sensor [108] which works based on photonic band gap principal. It has resolution 2 × 10^−6^ RIU in RI range 1.333 to 1.390. Demodulation technique was applied here. At RI 1.35 it shows a blue shift of 110 nm for RI change of 0.02 (Figure 39).

**Figure 40:**
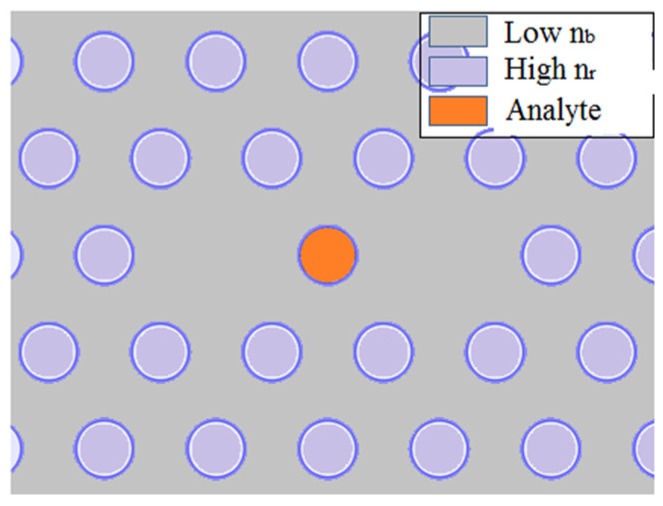
Dual core photonic bandgap fiber sensor; fabricated from a low index (n_b_) host material and higher index (n_r_) rod inclusions for analyte sensing (Figure courtesy from the reference [35]).

**Figure 41:**
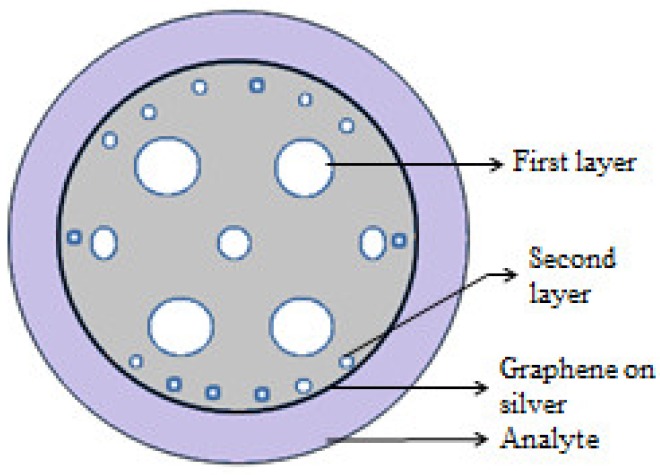
Cross-section of the proposed graphene-silver-based SPR sensor with external analyte channel (Figure courtesy from the reference [111]).

**Figure 42:**
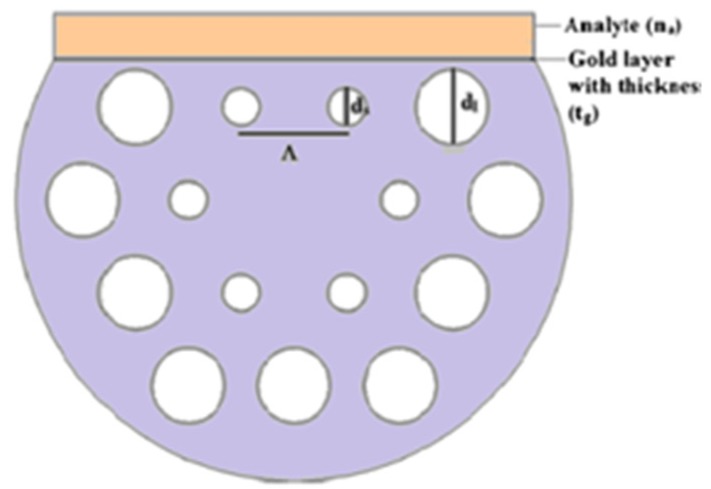
Cross-section of the proposed gold layer containing D-shaped PCF refractive index sensing probe which worked based on SPR theory (Reproduced from [33], with the permission of Springer publishing).

**Figure 43:**
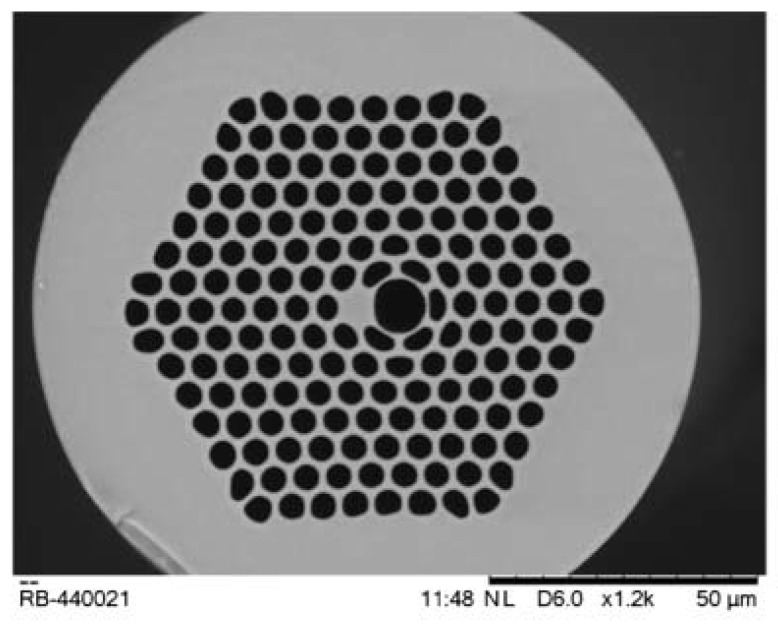
Selectively gold layer coated (around large cavity) birefringent PCF sensing probe used for RI measurement (Reproduced from [112], with the permission of OSA publishing).

Also different interferometry techniques are combined with PCF to make an advanced sensing system. Jha et al. demonstrated an interferometry based sensing probe in which a LMA PCF spliced between two single-mode fiber [109]. Length of the interferometer was 32–mm. This sensor has a high resolution of 2.9 × 10^−4^ in RI range 1.38–1.44. During this period hollow nature of PCF successfully combined with selective infiltration technique to make an improvised PCF sensing probe. Using this technique on a probe was reported [110], in which one air hole of a solid core PCF is filled with liquid. Here a strong field overlap takes place between core mode and mode associated with fluid infiltrated waveguide. Its sensitivity is 30,100 nm/RIU with resolution 4.6 × 10^−7^. A dual core PCF works based on mode coupling between two cores. A RI sensing probe was proposed [35] based on a dual core PCF in which central air hole is filled with suspected analyte (Figure 40). It shows sensitivity 7000 nm/RIU for a large RI variation. Sensitivity of PCF-based RI sensors enhanced multiple times when PCF is integrated with surface Plasmon resonance effect. Here, sensitivity can be determined from resonance peak shift. Dash et al. reported a graphene and silver coated birefringent PCF probe having external flow of analyte [111] for RI sensing. It has sensitivity 860 RIU^−^^1^ and resolution 4 × 10^−5^ RIU (Figure 41). Also, a gold layer coated D-shaped PCF probe was proposed [33] with high average sensitivity 7700 nm/RIU and resolution 1.30 × 10^−5^ RIU in refractive index range 1.43–1.46 (Figure 42). Recently, Rifat et al. successfully fabricated [112] a birefringent PCF-based selectively gold layer coated sensing probe (Figure 43) with sensitivity 11,000 nm/RIU for RI variation from 1.33 to 1.42. A few more RI sensors are presented in Table 4.

## 4. Limitations and Technological Advancement

At the very beginning PCF was applied as a waveguide. Then after nearly four years it started to be used as a sensor. So, PCF sensor technology is now at its infancy stage. In spite of that research progressed very rapidly in this filed due to its versatile and advanced optical properties over conventional optical fiber sensor and commercially available bulk sensors. Throughout this time many PCF sensors have been proposed and fabricated but it cannot be denied that the majority of the designed sensors are still at the proposal stage. At present the light of hope is the drastically developing PCF sensing technology. PCF was stated to be fabricated with a very popular stack and draw technique [2]. With this technique asymmetric, complex, and submicron structure fabrication was almost impossible. Then with time drilling [123], 3D printing [124], sol-gel [125], and extrusion [126] techniques were developed for the fabrication of advanced PCF. Selective infiltration of air holes either with analyte or particles [105,127] and application of noble metal or thin film coating inside air hole or outside of PCF [128,129,130] enhance its sensitivity several times over existing fiber sensors. Capillary force, focused ion beam milled micro channels can be applied to fill the air holes [131,132,133,134]. For a uniform and controlled noble metal coating chemical vapor deposition technique can be applied [135]. PCF sensors can be attached with any system due to its small size. Very small length of PCF is needed to make a sensing probe. If mass fabrication of a PCF sensor is possible then the cost of each sensor will be very low; they could even be used in household applications. Observing the exponential growth in PCF fabrication technology we are really hopeful about its industrial applications, detection of wide range of bio-chemical analytes, access of lab on a chip. Recently, for sensing in THz region some polymer like, TOPAS, poly (methyl methacrylate) (PMMA), polyamide-6 (PA6) bases PCF are designed [46,116,136,137]. Which is a great integration of fiber and THz technology.

## 5. Conclusions

Evolution of different type of PCF physical sensors are precisely discussed in this article. Starting from the interferometry based PCF sensors; design and fabrication of many PCF with advanced optical properties as well as their application in physical parameter sensing is summarized. This article starts with theoretical framework and basics of PCF and continues with the discussion of temperature, pressure, strain, twist, curvature, electromagnetic field, and refractive index sensors. Lastly it ended with a brief discussion on present concern and future hopes of PCF sensing technology.

From the above-discussion we can say that PCF sensor technology is a highly promising branch of modern optics and it has a lot of future possibilities. If PCF sensors wants to successfully compete with current commercially available techniques then it has to develop fabrication technology, enhance real time and industrial applications. At present scenario it is clear that many PCF sensors having advanced sensitivity showing their potential in wide range of sensing application with their very small size, robustness, flexibility, immunity against harsh environment, and many more. So, we can hope that PCF-based sensors will overcome their current limitations soon and prove suitable in large scale applications in industry as well as daily life. We are also hoping that this review article will give readers a clear idea about the current trends in the development of PCF physical sensors.

## Figures and Tables

**Table 1:** Comparative representation of different PCF-based temperature sensor.

Reported Structure	Sensing Temperature Range (°C)	Observed Quantity	Sensitivity	Ref.
TOPAS polymer optical fiber Bragg grating	21–33	Wavelength	−78 pm/°C	[46]
Tip interferometer using PCF spliced with SMF	20–100	Wavelength	10 pm/°C	[47]
Mach–Zehnder interference technique combined with PCF spliced with MMF	30–120	Phase shift/length	0.4272 radian/°C/cm	[48]
Fabry–Perot interferometer-based PCF containing inline microcavity and spliced with SMF	26–103	Wavelength	12 pm/°C	[49]
Plasmon resonance-based liquid crystal PCF containing gold nanowire	30–50	Wavelength	10 nm/°C	[50]
Microcavity incorporated solid core PCF concatenated with tapered SMF	40–80	Power	0.21 dBm/°C	[51]
Compact and liquid infiltrated asymmetric dual elliptical core PCF	30–34	Wavelength	42.99 nm/°C	[52]
Multibeam Mach–Zehnder interferometer using a PCF with two asymmetric cores	25–500	wavelength	1.24 pm/°C	[53]
Isopropanol-filled PCF long period grating	20–50	Wavelength	1.356 nm/°C	[54]
Selectively filled solid core PCF consisting a central air bore	−80 to 90	Wavelength	−6.02 nm/°C	[55]

**Table 2:** Comparative representation of different PCF-based pressure sensors.

Reported Structure	More about These Sensors	Sensitivity	Ref.
Periodically tapered long-period gratings combined with PCF	Can measure pressure up to 180 bar	11.2 pm/bar	[61]
Modal interferometer based high birefringence PCF	-	3.36 nm/MPa	[62]
Polarization-maintaining PCF-based Sagnac interferometer for downhole application	Measured at 1320 nm	4.21 nm/MPa	[63]
Side-hole polarization-maintaining PCF	-	−2.30 × 10^−5^/MPa	[64]
Bragg grating based highly birefringent microstructured optical fiber	Measured at 1550 nm	33 pm/MPa	[65]

**Table 3:** Comparative representation of different PCF-based strain sensors.

Reported Structure	Strain Range (µε)	Sensitivity	Ref.
PCF-based long-period fiber-grating	0–800	−7.6 pm/µε	[73]
PCF-based Mach–Zehnder type interferometers introducing coupling point	0–3250	~2.2 pm/µε	[74]
Fiber Bragg gratings photo-written in PCF having refractive index-neutral germanium/fluorine codoped core	0–3500	1.166 pm/με	[75]
In-line fiber Mach–Zehnder interferometer using solid core large mode area PCF	0–2500	−3 pm/µε	[76]
Modified PCF-based Mach–Zehnder interferometer	0–1300	11.22 dB/mε	[77]
Fiber ring cavity laser with a photonic crystal fiber PCF in-line Mach–Zehnder interferometer structure	0–2100	2.1 pm/µε	[78]
PCF with two asymmetric cores	0–4000	−1.59 pm/µε	[53]

**Table 4:** Collective representation of various PCF-based refractive index sensors.

Reported Structure	Spectral Range (nm)	RI Range	Observed Quantity	Sensitivity	Resolution (RIU)	Ref.
Stable photonic crystal fiber modal interferometer	1250–1340	1.33–1.45	Interference pattern shift	-	7 × 10^−5^	[113]
Surface long-period gratings incorporated D-shaped photonic crystal fiber	1250–1650	1.00–1.45	Wavelength	585.3 nm/RIU	-	[114]
Extrinsic cavity formed by a micromirror and a photonic crystal fiber tip which contains a bifunctional lens with large radius of curvature	1260–1350	1.328–1.357	Intensity	-	2.60 × 10^−5^	[115]
Directional coupler based on PCF polymer fiber	400–900	1.337–1.344	Wavelength	1.66 × 10^3^ nm/RIU	~2 × 10^−6^	[116]
SPR based multicore flat fiber	1000–1500	1.470–1.475	Wavelength	23,000 nm/RIU	4.35 × 10^−6^	[117]
Four channel containing PCF combined with gold wire	1600–2000	1.30–1.79	Wavelength	3233 nm/RIU	3.09 × 10^−5^	[118]
D shaped PCF combined with metamaterials	755–830	1.34–1.36	Wavelength	3700 nm/RIU	2.70 × 10^−5^	[119]
Gold nanowire consisting solid core PCF	600–1100	1.27–1.36	Wavelength	2350 nm/RIU	2.8 × 10^−5^	[120]
SPR based dual polarized spiral PCF	550–850	1.33–1.38	Wavelength	4600 nm/RIU	-	[121]
Dual core based microstructured optical fiber	500–900	1.35–1.51	Wavelength	7000 nm/RIU	7 × 10^−6^	[122]

## Abbreviations

PCF	Photonic crystal fiber
SC-PCF	Solid core photonic crystal fiber
HC-PCF	Hollow core photonic crystal fiber
NA	Numerical Aperture
TIR	Total internal reflection
SMF	Single-mode fiber
MMF	Multi-mode fiber
MOF	Microstructure optical fiber
FBG	Fiber brag grating
FP Cavity	Fabry–Pérot cavity
LPG	Long period grating
LMA	Large mode area
MZI	Mach–Zehnder interferometer
SPR	Surface plasmon resonance
LC	Liquid crystal
PBG	Photonic band gap fiber
DC-PCF	Dual core photonic crystal fiber
D-PCF	D shaped photonic crystal fiber
LC-PCF	Liquid crystal photonic crystal fiber
